# A Probabilistic and Syntactic Account of Variable Clitic Agreement in Spanish Double Object Constructions

**DOI:** 10.3389/fpsyg.2022.815432

**Published:** 2022-07-12

**Authors:** Gustavo Guajardo

**Affiliations:** Department of Language and Culture, UiT The Arctic University of Norway, Tromsø, Norway

**Keywords:** Spanish, clitic, agreement, language variation and corpus, double object constructions, Bayesian mixed effects, attraction effects, intervention effects

## Abstract

In Spanish clitic-doubling constructions, the clitic should agree in number with its coreferential doubled noun phrase. However, the present corpus analysis with data from 21 Spanish varieties reveals that, under certain structural configurations, number agreement is not always realized on the third-person dative clitic. In fact, the data shows that non-agreement appears to be the norm when the indirect object is a lexical noun phrase (77 vs. 23%). In this paper, I investigate two possible explanations for this phenomenon: (i) a processing account *via* an attraction effect and (ii) a syntactic account based on intervention effects. These two hypotheses make clear and testable predictions that I examine by means of conditional inference trees and Bayesian generalized mixed-effects logistic regression modeling. The results of the statistical analyses are incompatible with an intervention account because this type of phenomenon is not sensitive to semantic features of the intervening element or to the true controller of agreement. Thus, I propose that the data is best analyzed as the interplay between attraction and the morphosyntax of the unmarked. In Spanish, this results in attraction effects from the DO in the unmarked word order and inanimate IOs showing a sort of differential dative marking, where animate IOs show a preference for full agreement. The findings reported herein show evidence of a complex and highly dynamic agreement mechanism of the clitic and highlight the probabilistic nature of morphosyntactic processes.

## Introduction

Agreement is a pervasive linguistic phenomenon whereby a dependent phrase agrees in certain features with an agreement controller (Corbett, 2006). For example, in *John visits his parents on the weekends*, the NP *John* controls agreement on the verb *to visit* so that it is realized as *visit-s* and not *visit.* Naively, one would think that if agreement is obligatory in a language then agreement should be realized whenever its conditions are met. However, there is plenty of evidence that this is not always the case. For example, by studying the effect of intervening elements between the controller and the dependent element researchers have found that agreement mechanisms are indeed subject to interfering or attraction effects with the result that the expected agreement relationship may not be properly established (more on this in Section “SPANISH CLITIC DOUBLING”).

Interestingly, however, lack of obligatory agreement does not always lead to ungrammaticality, contrary to what one might assume. In Icelandic raising constructions (1a), for example, agreement between the verb and the nominative subject NP can be blocked by an intervening dative experiencer as in (1b), but the sentence is not rendered ill-formed; the language resorts to default third-person agreement in a sort of repair mechanism to salvage the sentence.


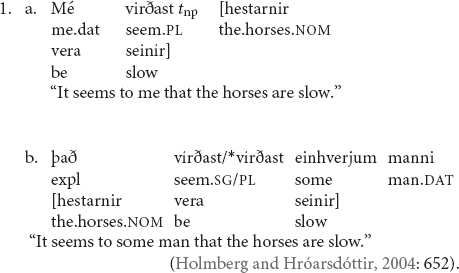


In (1a), the verb *virðast* “they seem” agrees with the nominative NP *hestarnir* “the horses.” In (1b), however, there is an intervening dative NP between the verb and the nominative NP, which prevents the establishment of agreement, resulting in singular (default) agreement on the verb. But it is not always the case that failure to establish agreement results in grammaticality. In French, an intervening lexical dative experiencer also blocks agreement between the verb and the subject NP but the sentence becomes unacceptable (2a); removing the dative experiencer and replacing it with a clitic salvages the sentence (2b).


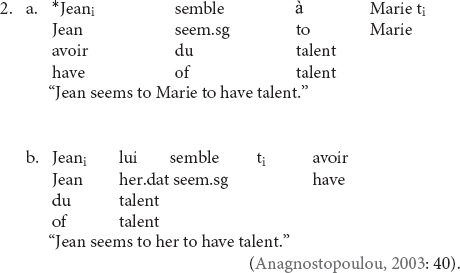


In (2a), the dative NP à *Marie* “to Marie” acts as an intervener between the verb *semble* “seem” and the subject NP *Jean*, rendering the sentence ill-formed. When à *Marie* is removed (and replaced with a clitic), agreement can be established, and the sentence is salvaged.

Lack of obligatory agreement is not restricted to subject-verb agreement. All types of agreement dependencies could in principle be subject to it. For example, even though Spanish requires adjectives to agree with the noun they modify in gender and number, the following examples demonstrate lack of obligatory agreement between the adjective and the head noun after the copula^1^.


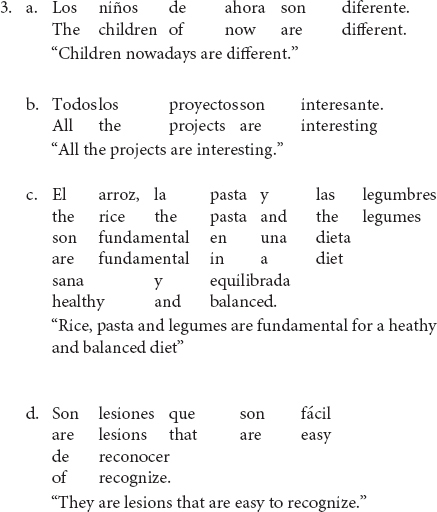


In (3a-d) the adjectives *diferente* “different,” *interesante* “interesting,” *fundamental* “fundamental” and *fácil* “easy” refer to a plural subject. Both the NP subjects and the copula show plural agreement, yet the adjective appears in its default singular form^2^. Note that these adjectives do not inflect for gender, so the agreement mismatch only applies to number agreement.

In this paper, I examine another phenomenon in Spanish where lack of obligatory agreement does not lead to ungrammaticality, but to default agreement similar to the Icelandic case in (1). More specifically, this paper is concerned with agreement between a clitic and its doubled NP in Spanish clitic-doubling constructions with third-person dative clitics (4).


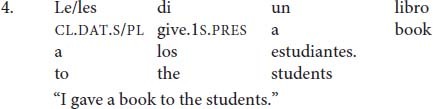


In (4), the dative clitic can appear in the singular *le* or the plural *les* even though its referent *los estudiantes* “the students” is plural.

Descriptively, I show that agreement in certain clitic-doubling configurations is most often left unrealized and this is true across all Spanish varieties (contra what has been suggested in previous literature). Analytically, I study the linguistic factors that can account for the (un)realization of agreement between the clitic and its coreferential NP and show that there are three main predictors of (lack of) agreement, namely the animacy of the lexical indirect object, the number features of the direct object and word order. I discuss the findings in the context of attraction versus intervention phenomena and argue that the evidence aligns better with an attraction account^3^.

## Spanish Clitic Doubling

There is a vast literature on clitic doubling in Spanish but also in Romance and more generally across languages. In this section, I will focus on those aspects of clitic doubling that are relevant for the understanding of variable clitic agreement in double-object constructions (for a detailed overview of clitic doubling see Anagnostopoulou, 2017).

Clitic doubling can be defined as the phenomenon where a phonologically bound element (i.e., the clitic) expresses agreement features of an NP that is an argument of the verb in the same propositional structure (Adger, 2003; Harizanov, 2014; Anagnostopoulou, 2017). This means that the clitic and the co-indexed NP share the same case and phi-features. Languages differ in what types of NPs can be doubled and/or which clitics can appear in clitic-doubling constructions. For example, in Spanish doubling of the indirect object is almost always grammatical regardless of the type of IO (definite, indefinite, etc.) (Suñer, 1988; Roca, 1992; Torrego, 1995; Dufter and Stark, 2008) whereas the doubling conditions of the direct object are much more constrained and subject to dialectal variation to a greater extent than the IO clitic (Jaeggli, 1982; Suñer, 1988).

Focusing on the doubling of the indirect object, the following generalizations can be made: doubling of the IO is always possible but not required (5) except with left-dislocated NPs and strong pronouns. In these two cases, doubling of the IO is obligatory (6).


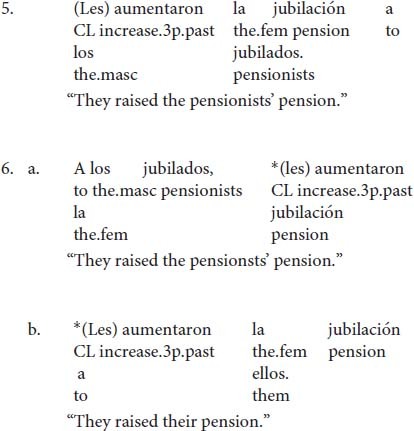


In (5) the IO can be optionally doubled, with the doubled option being the less marked form in contemporary Spanish (Becerra Bascuñán, 2006; Company Company, 2006; but see Hentschel, 2013 for a different conclusion). In (6a), the indirect object has been left-dislocated and therefore clitic-doubling becomes obligatory. Likewise, in (6b) clitic doubling is the only option because the indirect object is realized as a strong pronoun.

The analysis of the obligatoriness of clitic doubling with left-dislocated elements has been ascribed to the non-argumental status of the left-dislocated NP. It is generally assumed that it is base generated in this position (Roca, 1992). Under this analysis, a clause with a left-dislocated indirect object and a clause without an indirect object altogether are structurally equivalent. The question then is what occupies the argument position of the verb. Two possible analysis have been provided. In one analysis the argument position of the verb is filled with *pro* (Suñer, 1988; Torrego, 1995). Alternatively, the argument position is filled with a silent copy of the clitic and the clitic is the only argument of the verb (Roca, 1992). Regardless of the type of analysis of which empty category fills the argument position of the verb, the key aspect of these two accounts is that the argument position is phonologically empty and therefore the clitic carries the only visible phi-features of the indirect object.

The account of why strong pronouns must be doubled has also been subject to different interpretations. An important feature of Spanish strong pronouns is that they are always construed as [ + human] when in non-oblique positions (i.e., subject, direct and indirect object) (Strozer, 1976). Torrego (1995) distinguishes clitics that refer to animate NPs versus those that have an inanimate referent. She claims that only inanimate clitics occupy an argument position. Suñer (1999) follows Torrego’s analysis and assumes the argument position of the verb is always filled with *pro* when the clitic doubles a strong pronoun. For this author, the obligatoriness of clitic doubling of strong pronouns is triggered by well-formedness conditions for LF representation and interpretation. These conditions are dictated by Diesing’s (1992) Mapping Hypothesis, which states that syntactic trees are divided into two domains: the VP providing the domain of existential closure and the IP the restrictive clause. For pronouns, which are normally definite, so cannot be interpreted existentially, this means that they must move out of the VP. Suñer (1999) argues that clitic doubling of strong pronouns serves the purpose of complying with the Mapping Hypothesis because part of the chain between the clitic and the strong pronoun is outside the domain of existential closure before Spell-out.

For the problem at hand, the takeaway message is that neither left-dislocated indirect objects nor strong pronouns occupy the argument position, and therefore there is no overt constituent, other than the clitic, that bears the phi-features of the NP. In addition to this generalization, strong pronouns in Spanish are (+human) unless they are the object of a preposition. These two ingredients will be key to explaining the constraints on default agreement in Section Default Agreement and *pro*.

With this theoretical background in place, I will now introduce in more detail the phenomenon the present paper is concerned with.

## The Linguistic Phenomenon Under Investigation

As discussed in the previous section, in Spanish double-object constructions, the indirect object is typically doubled with a dative clitic, which must agree in number with it (7–8).


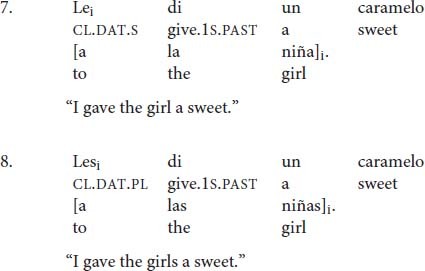


In (7) the indirect object NP *la niña* “the girl” is singular so the singular clitic *le* is required, whereas in (8) the plural clitic *les* appears because the indirect object *las niñas “*the girls” is plural.

Most research on clitic-doubling constructions has focused on the licensing mechanism of the phenomenon, whether it is obligatory or optional, and the semantic interpretation of clitic-doubled constructions as compared to structurally similar, but non-clitic-doubled, ones. A property of this phenomenon that has received little attention, however, is the fact that number agreement between the clitic and its coreferential NP does not always obtain, such that a *singular* dative clitic may co-occur with a coreferential *plural* indirect object (9)^4^.


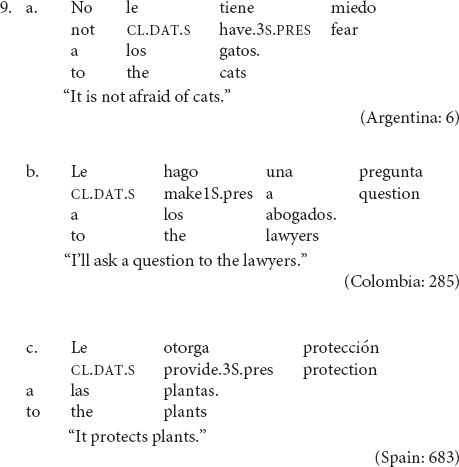


In all of the examples in (9), the dative-doubled NP is plural but the clitic appears in the singular form *le.* Roca (1992) calls this phenomenon *defective agreement*, that is, the use of a third person singular dative clitic when the apparent “agreeing” element is clearly plural. I will refer to it with a more neutral term and simply call it *default agreement*. While it has been acknowledged in the literature that this phenomenon exists (e.g., Kany, 1951; DeMello, 1992; Franco, 1993; Torrego, 1995; Belloro, 2007), it seems that researchers have assumed that this type of agreement mismatch is possible in any construction in which the clitic occurs, that it is a somewhat rare phenomenon, and subject to dialectal variation (i.e., not present in all varieties). In this paper, I show (i) that the structural context in which default agreement can occur is highly constrained, (ii) that default agreement is far more frequent than full agreement when the appropriate structural conditions for it are met and (iii) that this is true across all Spanish varieties. Furthermore, the data and analysis in this paper ultimately provide further support for the analysis of the dative clitic as an (object) agreement marker according to Preminger (2009)’s diagnostics (more on this in Section “Discussion”).

## Constraints on Default Agreement

As I alluded to in the Introduction, default agreement is highly constrained, and its optionality is only apparent insofar as the structural configurations in which it can appear. In this section, I present the contexts that allow and disallow default agreement (the contexts will be expanded once the corpus data has been analyzed). The picture that emerges is that this type of default agreement is structure-dependent and only possible when the doubled NP is in argument position.

Default agreement is only possible in clitic-doubling constructions (10a), but it is not possible when the clitic stands alone without an overt coreferential NP (10a′)^5^. Default agreement is also disallowed in left-dislocated constructions (11). With dative experiencers, however, the singular clitic can occur but full agreement seems to be preferred (12)^6^.


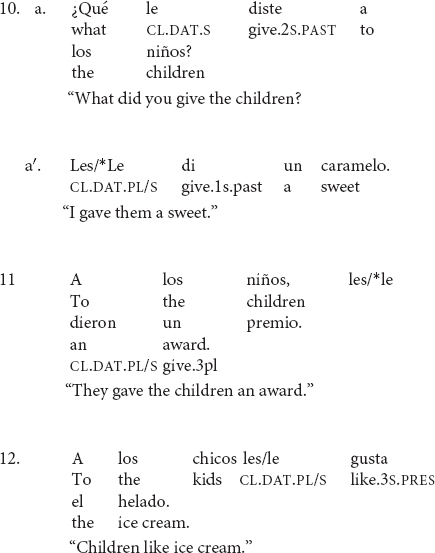


The data in (7) illustrate that default agreement is only possible in clitic-doubled sentences as in (6a). If the co-referential NP is not overtly realized as in (7b), then full agreement seems to be the only option. However, clitic-doubling is a necessary but not sufficient condition. In (8), there is clitic doubling of the left-dislocated dative NP, but default agreement is ungrammatical. (12) contains a dative experiencer, which is also doubled, but there is a strong preference for full agreement. The rest of the paper will focus on double-object constructions, so I will leave dative experiencers aside for future work.

The data in (10--12) suggest that default agreement is highly constrained with respect to the structural contexts in which it is allowed. Importantly, not every construction with a dative clitic allows for default agreement^7^. The question then becomes what allows default agreement to take place, and conversely, what disallows it. To answer this question, I will distinguish cases where agreement is categorical as in (10a′) and (11) and cases where default agreement is preferred as in (10a). After all, full agreement is always allowed and never ungrammatical, thus default agreement is a highly preferred alternative to full agreement but not the only grammatical option. In this respect, it is different from the dative intervention effects found in some varieties of Icelandic where full agreement is not possible whenever a dative NP intervenes between the nominative subject and the verb (Holmberg and Hróarsdóttir, 2004; Sigurðsson and Holmberg, 2008).

The generalizations above raise two related questions. The first question concerns the variability of the phenomenon, namely which factors favor default agreement in those contexts in which both full and default agreement are possible. The second refers to the syntactic constraints governing default agreement, that is, why default agreement is allowed in some clitic constructions but not in others. To answer the first question, I will use corpus data and statistical modeling. The answer to the second question requires a more theoretical analysis based on what we has been found about clitics and the syntax of doubled elements discussed in Section “Spanish Clitic Doubling.”

I will now briefly discuss two different processes that have been proposed to account for lack of obligatory agreement across languages, so that we can then determine which of the two processes best accounts for our data.

## Types of Agreement Failures

In this section, I will discuss two types of agreement failure that are found in the literature. One type is called attraction effects (Bock and Miller, 1991) and the other intervention effects (Holmberg and Hróarsdóttir, 2004; Sigurðsson and Holmberg, 2008; Preminger, 2009). By the end of the section, I will lay out the type of evidence that can help us decide what type of phenomenon is exemplified in our case of default agreement.

Although both mechanisms refer to instances in which agreement is not “properly” established, they refer to qualitatively different phenomena. The former is mostly associated with processing difficulty due to an intervening candidate that tricks the parser under certain conditions. In other words, agreement failure due to an attraction effect is normally considered an error and lies outside the grammar. The latter type of agreement failure refers to a syntactic configuration where an intervening element blocks agreement from happening due to structural reasons. Unlike attraction effects, intervention effects are part of the grammar and not due to speakers’ errors.

### Intervention Effects

Icelandic and Basque have both been reported to display intervention effects in different structures. In Icelandic dative-nominative constructions, the third person nominative NP normally controls agreement (12a) but if the dative object intervenes between the nominative NP and the verb agreement may be blocked (subject to dialectal variation) with the result that the verb may surface in default third person singular (12b) (Sigurðsson and Holmberg, 2008).

Similarly, Preminger (2009) shows that, in some Basque varieties, a dative NP can act as an intervener in ditransitive constructions as in (13).


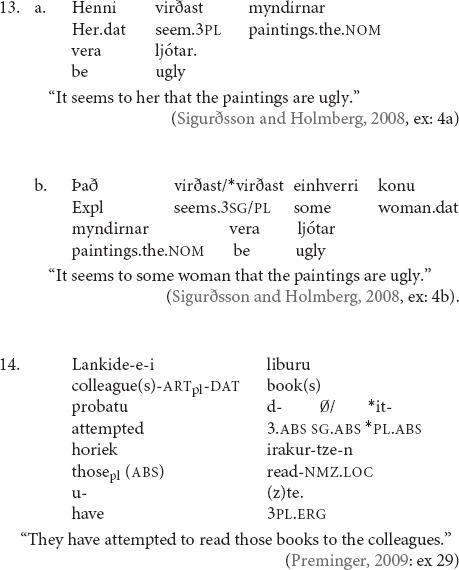


In (13), the dative NP *lankideei* “the colleagues” blocks agreement between the absolutive NP *liburu horiek* “those books” and the auxiliary *duzte*, which only carries default singular agreement but does not agree in number with the plural absolutive NP.

Sigurðsson and Holmberg’s (2008) analysis of the Icelandic data is that person and number are separate probes. Important for their analysis is the fact that agreement of the verb with 1^st^ and 2^nd^ person nominative is always banned in the dative-nominative construction. In this case, default third person is the only option regardless of dialect. The differences between a dialect that allows agreement in the presence of an intervening dative object and a dialect that does not is in the timing of the derivation. In dialects that allow agreement number probing of T takes place after raising of the dative out of the VP, whereas in a dialect where agreement is not possible T-raising to number happens prior to dative raising. In the latter configuration, dative will always intervene thus blocking number agreement between the verb and nominative.

The goal of Preminger (2009) is different in that he uses the data to propose a diagnostic to distinguish between clitics and agreement morphemes, so his main interest is not in accounting for the intervening effect *per se*. Having said this, at the end of the article he offers a possible implementation of his analysis in terms of the person-case constraint (PCC). To do this, he adopts Sigurðsson and Holmberg’s (2008) proposal that person and number are separate probes and assumes that the dative argument is in the specifier position of an Applicative phrase. Based on Anagnostopoulou (2003) and Béjar and Rezac (2003) account of the PCC, he assumes that dative NPs prevent Person from probing further but do not value the person feature with their own person feature. Thus, the presence of a dative argument in the specifier position of ApplP results in default person features. Next, when Person and the dative argument are clausemates, the dative argument is doubled with a clitic in Basque and this process renders the dative NP invisible to further Agree operations. Valuing of the number feature is carried out by *v* but, since the dative argument is invisible, the probe must keep searching until it finds the theme, which values the number features on *v.* Crucially, when Person and the dative argument are not clausemates, there will be no clitic doubling. Thus, the dative argument will still be visible to Agree when *v* probes for number. But, as with person, datives cannot value a probe with their own phi-features resulting in default person and number agreement.

These two analyses suggest that the only relevant factor for intervention is the presence of an element that lies between the probe and the goal. From this, it appears that the number feature of the intervening element is not important in the blocking effect associated with default agreement on the verb. This makes certain predictions with respect to the phenomenon under analysis that I will present at the end of this section.

### Attraction Effects

Villata and Franck (2020) identify three main findings in the literature on attraction in sentence production. The first of these is that attraction is modulated by semantic factors. Thus, grammatically singular but notionally plural nouns generate more plural verbs than notionally singular nouns (Eberhard, 1997; Haskell and MacDonald, 2003; Bock et al., 2004). The next finding is that attraction appears to be asymmetric in the sense that it is stronger for plural attractors than for singular ones (e.g., Bock and Miller, 1991; Eberhard, 1997; Hartsuiker et al., 2003; Wagers et al., 2009). The third factor is that attraction is modulated by the hierarchical position of the attractor more than by its linear position (e.g., Bock and Miller, 1991; Vigliocco and Nicol, 1998; Franck et al., 2002, 2006).

Although most of the literature on attraction effects has looked at subject-verb agreement, there is a growing body of research looking at other types of agreement dependencies such as antecedent-pronoun agreement. The findings in this area are still somewhat inconclusive as different studies have yielded conflicting results. For example, some studies have found that antecedent-pronoun agreement seems to be as sensitive to attraction effects as subject-verb agreement (Bock et al., 1999, 2004; Patil et al., 2016) whereas others have found that gender agreement in antecedent-reflexive dependencies is not affected by the interference from other competing antecedents (e.g., Nicol and Swinney, 1989; Sturt, 2003; Dillon et al., 2013).

In a recent study, Jäger et al. (2020) replicate the experiment in Dillon et al. (2013) with a higher number of participants (40 vs. 181). The main conclusion of their study is that they find no difference between attraction effects in subject-verb agreement and antecedent-reflexive dependencies. The caveat to this finding, however, is that the dependent measure used in Dillon et al. is total fixation time. The authors highlight that total fixation time has the potential to be too broad a measure because it subsumes all types of dependent measures from eye-tracking. To explore whether a more fine-grained analysis could shed some light on the status of reflexives in attraction effects they analyze first-pass reading time and first-pass regressions. They find no effect of first-pass reading time in the interaction between type of dependency and attraction effect (i.e., neither subject-verb agreement nor reflexives showed attraction effects). In first-pass regressions, they find an asymmetry between grammatical and ungrammatical sentences. In grammatical sentences reflexives show inhibitory interference (i.e., slowdown in reaction time) whereas in ungrammatical sentences subject-verb agreement shows a clear interference effect but reflexives do not show any interference. Thus, it remains an empirical question whether, and to what extent, attraction effects are mediated by the type of linguistic dependency or whether all types of agreement relations can, in principle, be subject to attraction effects.

At this point it is worth comparing these findings with the characteristics of default agreement as a way to help the reader understand the relationship between the two. The first thing to highlight is that the phenomenon exemplified in default agreement of the clitic is one where the supposedly attractor is singular which causes an attraction on the clitic over the plural controller NP. Thus, default agreement appears not to follow what the majority of the attraction effects have shown. But, this in itself is not enough to discard attraction as a possible explanation. On the one hand, it is not impossible for singular attractors to trigger attraction, it is just less likely. But most importantly, most studies on attraction effects that have come to this generalization have studied subject-verb agreement, which is qualitatively different from both object agreement and clitic-antecedent concord. A relevant study concerning this difference is Santesteban et al. (2017) where they look at attraction effects between a left-dislocated NP and a following accusative clitic. In line with other studies that found no attraction effect between a reflexive and its antecedent (Dillon et al., 2013; Parker and Phillips, 2017 but see Jäger et al., 2020 for a different result), they find that in a self-paced reading task the antecedent-clitic relation is not sensitive to attraction effects. In a second task, where they use event related potentials (ERPs), they find that the type of response is different from what has been found with subject-verb agreement attraction effects. More specifically, they find a negative ERP component whereas subject-verb agreement effects have been shown to elicit a late positive P600 (Osterhout et al., 1996; Nevins et al., 2007; Frenck-Mestre et al., 2008).

Hence, it is not impossible to conjecture that the asymmetry found for subject verb agreement may simply not hold for clitic agreement because they are a different type of agreement phenomena. Ultimately, this is an empirical question. In this paper, I will assess whether we are in the presence of an attraction effect by looking at whether there is an interaction between the number of the DO and the order of both objects with respect to each other (i.e., DO-IO vs. IO-DO).

**FIGURE 1:**
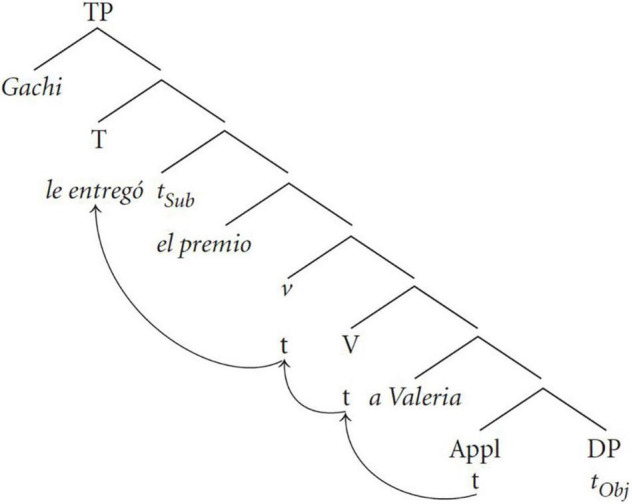
Syntactic structure for Spanish double object constructions (Cuervo, 2003: 229).

Another important issue in attraction effects is whether it is the linear distance between the clitic and the DO that matters or whether what is important is the hierarchical position of the clitic with respect to the DO and the IO. As I mentioned above, attraction effects appear to be very sensitive to the hierarchical structure more so than to linear order. This question is closely related to the point in the derivation where agreement is computed. To look at this question more closely, we need to first have a syntactic structure for the double object construction. Figure 1 shows the syntactic tree proposed in Cuervo (2003). In this structure, the clitic is the head of an Applicative Phrase, the lexical IO is in the specifier position and the complement of AppP is filled with the lexical DO.

Two things are worth pointing out about this structure. First, in the structure before movement, the clitic and the DO will always be closer to each other than the clitic to the lexical IO (Shen et al., 2018)^8^. Given this observation we do not predict that surface word order should matter if (default) agreement is established before movement because the relationship and distance between the clitic and the objects will always be the same. On the other hand, if agreement is computed after movement then we might expect that the surface word order of the two objects will matter. More concretely, we would expect that default agreement should be more likely with the word order DO-IO because the DO is first in the search domain of the clitic. Conversely, when the order of objects is reversed, we would expect that default agreement should be less likely.

One way to test whether it is the linear order or the hierarchical position of the clitic and the object that is important for default agreement is to examine whether the distance between the DO and the clitic, and the clitic and the IO plays a role in favoring or not default agreement. If it does, then it is more likely that it is linear distance, and not hierarchical structure, that is involved in the realization of default agreement. Conversely, if linear distance is not predictive of default agreement then this would suggest that what matters is the hierarchical relationship between the clitic and the lexical objects.

Having presented the two types of agreement failures, I will now outline the hypotheses and predictions that will help us determine whether default agreement is best explained as a case of attraction or intervention.

### Hypotheses and Predictions

The discussion in the previous section helped us understand the two different phenomena that have been discussed in the literature as cases of agreement failures. It also made it clear that they make very different predictions regarding the factors that may or may not be predictive of defective agreement. Thus, the following hypotheses and predictions will be tested.

Hypothesis I: Default agreement can be best accounted for as a case of attraction.

Hypothesis II: Default agreement is a by-product of the hierarchical order of the two objects.

Hypothesis III: The animacy of the objects will be predictive of default agreement.

Hypothesis I refers to the two cases of agreement failure that we discussed. Hypothesis II is concerned with the relationship between the objects and the clitic and it aims to test whether it is linear distance or hierarchical structure that characterizes default agreement. The third hypothesis is based on other areas of Spanish grammar where animacy plays a crucial role in morphosyntax such as in differential object marking, clitic doubling of DOs and the availability of strong pronouns. The three hypotheses make the following predictions:

Prediction 1: If default agreement is a case of an attraction effect, then it is predicted that the number feature of the direct object will be a significant factor in favoring default agreement. More concretely, it predicts that singular DOs should favor default agreement to a larger extent than plural DOs. On the other hand, if default agreement is a case of intervention, then the number feature of the object should not matter. Under an intervention account, only the position of the objects should be relevant such that the DO IO word order should prefer default agreement but, crucially, we should find no interaction between word worder and the number of the DO.

Prediction 2: Linear distance (counted in syllables) should not be predictive of default agreement if hierarchical structure determines default agreement. In addition, we should find evidence in favor of word order such that the unmarked order DO IO should favor default agreement because, although the clitic c-commands both objects, the DO is first in the search domain. Conversely, the inverse worder IO DO should favor full agreement^9^.

Prediction 3: Inanimate indirect objects should favor default agreement because inanimate nominals in Spanish require less marking than animate ones such as differential object marking and clitic-doubling of DOs.

With the theoretical background and the hypotheses and predictions in place, in the next section I introduce the present study.

## Methodology

In this section I describe how the data was extracted from the corpus, the predictor variables and the statistical analysis used to study default agreement.

### The Data

The data was extracted from the Web/Dialect version of Corpus del Español (Davies, 2016). The corpus contains nearly 2 billion words from 21 Spanish-speaking countries, including the United States^10^ ′^11^. The data is tagged for morphosyntactic, lexical and semantic information and comes from websites, including written blogs and forums. Thus, the register is quite informal, which makes it a great tool to study non-standard features of language. The source of each sentence, whether it is a website or a blog, is included in the results of the search as is the website’s address of each sentence. Data extraction was done manually on the corpus web interface alternating the position (*pre-* or *postverbal*) and the number of the clitic (*sg* vs. *pl*), the gender, number and definiteness features of the DO (*masc* vs. *fem*; *sg* vs. *pl*; *bare* vs. *definite* vs. *indefinite*) and the gender of the IO (*masc* vs. *fem)*. In addition, the relative order of the objects was also manipulated so that both possible word order were extracted, namely DO IO and IO DO. Only sentences with definite IOs and with a token frequency of at least 2 were extracted.

After manual removal of duplicates, a total of 2,414 sentences were left for analysis which were coded for the following variables: FINITE VERB, NON-FINITE VERB, PERSON, NUMBER OF VERB, ANIMACY OF DO, ANIMACY OF IO, POSITION OF CLITIC, NUMBER OF DO, GENDER OF DO, GENDER OF IO, DEFINITENESS OF DO, TYPE OF IO, WORD ORDER, DISTANCE CLITIC TO IO, DISTANCE CLITIC TO DO. Three more variables were included that are automatically generated by the corpus and these are SOURCE, COUNTRY and WEBSITE. A summary of all the variables and the levels of each variable are presented in Table 1.

**TABLE 1:** Coded variables and their possible values.

Variable name	Levels	Variable name	Levels
SOURCE	B(log), G(eneral)	POSITION OF CLITIC	Pre, Post
COUNTRY	All 21 countries	NUMBER_DO	SG, PL
WEBSITE	Website address	GENDER_DO	Fem, Masc
FINITE_V	Any verb	GENDER_IO	Fem, Masc
NON-FINITE_V	Any verb	DEFINITENESS_DO	Def, Indef, Bare
PERSON_V	1st, 2nd, 3rd	TYPE_IO	Pron, NP
NUMBER_V	SG, PL	WORD ORDER	DO-IO, IO-DO
ANIMACY_DO	High, Low	DISTANCE CL TO DO	Continuous
ANIMACY_IO	High, Low	DISTANCE CL TO IO	Continuous
FIRST ELEMENT	Consonant, Vowel		

All variable levels are quite uncontroversial and do not necessitate an explanation of how they were coded as they refer to grammatical features that require no judgment on the part of the researcher. The exception to this generalization is animacy, which involves a certain degree of subjectivity as to what constitutes an animate or an inanimate noun. Thus, to code this variable in a systematic way I adapted Bresnan et al.’s (2007) system of four animacy levels: “human,” “organization,” “animal/intelligent machine” and “inanimate.” Since there is psycholinguistic evidence that not all animals are treated in the same way by speakers (Radanović et al., 2016), I coded “dogs” and “cats” in the same category as human beings^12^. Consequently, the four levels of animacy were High (humans, dogs and cats), Mid-High (organizations), Mid-Low (animals and intelligent machines), Low (inanimates). However, after fitting the statistical models, it was clear the difference was only between nouns high in animacy and the rest, so I decided to binarize this variable and collapse all non-high levels (mid-high, mid-low, low) into a single low animacy level.

### Statistical Analysis

The statistical analysis was performed in R 4.0.3 (R Core Team, 2016). Two complementary analyses were performed. In the first analysis, I used a conditional inference tree in the party package (aka classification trees), a machine learning algorithm that is very easy to interpret. The second statistical model is a Bayesian mixed-effects logistic regression. In what follows I describe the models and the steps that went into model selection for the mixed-effects model.

Conditional inference trees (Hothorn et al., 2006) are a non-parametric type of model that allows the user to simply enter the predictor variables without interactions and the algorithm finds significant interactions on its own (if there are any) and displays them in the form of a tree, so it is very easy and reader friendly to spot the interactions found by the model. Conditional inference trees belong to a class of statistical models that use recursive partitioning as the main algorithm. Informally speaking, the algorithm first tests if any of the independent variables are associated with the response variable. If it finds more than one independent variable that is associated, then the model determines the strength of association of each of the independent variables with the response variable. The variable with the strongest association is selected for the first binary split. For example, if the independent variable is binary with values M and F, then one subset will contain all the observations with value M and the other subset will contain all those with value F. Each subset constitutes a branch in the tree. This procedure is recursively repeated until all independent variables have been evaluated.

From the point of view of the analyst, classification trees are a useful tool to detect possible interactions that can be entered into a more complex model such as mixed-effects models. Another advantage of classification trees is that they can show very complex interactions that would be very difficult to model (let alone interpret) in a logistic regression. Conditional inference trees have the additional advantage over regular classification trees, such as those in the rpart package, that they have been designed to avoid both overfitting and the bias of regular classification trees toward covariates with many possible splits or missing values. Thus, they are a good complement to logistic regression which assumes a linear relationship between the outcome and the predictors.

Based on the results of the classification tree, a Bayesian mixed-effects logistic regression model was fit with two random intercepts COUNTRY and VERB. The website addresses were not included in the final model because there were very few websites that appeared more than once, which means that nearly every single address was associated with only one of the clitics and therefore the model would overfit.

The Bayesian models were fit using the Stan modeling language (Carpenter et al., 2017) with the brms package (Bürkner, 2017). Four sampling chains ran for 6,000 iterations each with a warm-up period of 3,000 iterations, thereby resulting in a total of 12,000 samples for each parameter tuple. For the fixed-effect priors, I followed Gelman et al. (2008)’s recommendation to use Cauchy priors with center 0 and scale 2.5 for the coefficients and a Cauchy prior with center 0 and scale 10 for the intercept. This loosely constrains the parameter effects to range between –2.5 and 2.5 while allowing for larger values if there is enough evidence for that in the data. For the random effects, I used the default priors, namely a Student’s *t-*distribution (*v* = 3, μ = 0, σ = 2.5).

Within a Bayesian inference framework, there is no consensus when it comes to the best method for hypothesis testing and there is still ongoing debate about the pros and cons of the different methods available. Some researchers use the 95% credible interval of a fitted model to test whether it contains zero and if it does not, then they conclude the parameter has an effect different from zero. The problem with this approach is that it does not answer the question of how much evidence for an effect we actually have (Schad et al., 2021). Thus, in this paper I will use a combination of different sources of evidence to determine whether the effect of a predictor is different from zero. More specifically, I will use Bayes factors and the probability of direction.

Bayes factors are not without problems as they are very sensitive to the data and prior specifications. To mitigate this issue and ensure the results from the Bayes factors are reliable, I used orthonormal contrasts (Rouder et al., 2012; Makowski et al., 2019a,b) and calculated them three times (Schad et al., 2021). The Bayes factor allows us to calculate the probability of rejecting the null hypothesis of no effect for each parameter given the data. In other words, a Bayes factor tells us, based on the data and the model priors, how much we need to update our relative beliefs (Schad et al., 2021). The interpretation of Bayes factors is as follows (Jeffreys, 1961): BF < 1 evidence in favor of the null hypothesis (the parameter does not contribute to explaining the outcome). BF < 1 there is evidence against the null hypothesis. BF = 3–10 there is moderate evidence, BF = 10–30 there is strong evidence, BF = 30–100 there is very strong evidence and BF > 100 extreme evidence.

The probability of direction is an index of effect existence and it ranges from 50 to 100%. This value represents the certainty that an effect goes in a particular direction (negative or positive). The advantage of this index is that it is model independent in that it is only based on the posterior distribution and it does not depend on the scale of the response variable or the predictors. Another interesting property of the probability of direction index is that it is highly correlated with *p*-values, making it more interpretable for readers who are not familiar with Bayesian statistics (Makowski et al., 2019a,b). The difference between this index and the Bayes factor is that it does not measure the magnitude and importance of an effect. An effect can have high certainty of being positive (e.g., 99%) but the effect can be very small and close to zero. Values of 97.5 and 99.5% correspond to *p*-values of 0.05 and 0.01, respectively^13^.

The mixed-effects model was first fitted on all the variables selected by the conditional inference tree with two- and three-way interactions together with VERB, COUNTRY, DIRECTOBJECT and INDIRECTOBJECT as random intercepts. Different random slopes were evaluated by means of Bayesian leave-one-out-cross-validation (LOO-CV) (Vehtari et al., 2017) and model stacking (Yao et al., 2017). A model with random slopes on NUMBERDO and ANIMACYIO was chosen by both methods. With this full model, Bayes factors were calculated to determine the evidence in favor of rejecting the null hypothesis that the predictor variables had no effect on the outcome. Based on the results of the Bayes factor analysis, a smaller model was fitted with only those parameters whose Bayes factor was equal to or higher than one^14^. To ensure the smaller model was a better fit than the full model, the two models were also compared using LOO-CV and model stacking. Herein I report the results from the smaller model, as both LOO-CV and model stacking coincided in selecting the smaller model as the better fit.

The assessment of the final model consists in calculating Bayes factors and the probability of direction of each parameter to determine the magnitude and certainty of their effects.

## Results

This section presents the results of the descriptive statistics and the statistical models. I will start off by summarizing and describing the distribution of some of the most important variables and then I will present the conditional inference tree followed by the Bayesian mixed-effects logistic regression.

### Descriptive Statistics

**FIGURE 2:**
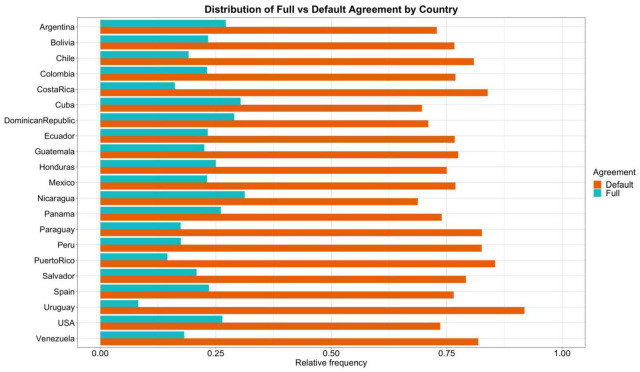
Distribution of full and default agreement by country. The x-axis represents the relative frequency of each clitic in each country.

Of the total 2,414 sentences, 1,855 sentences displayed default agreement and 559 full agreement, which results in a relative frequency of 0.77 and 0.23, respectively. The relative frequency of agreement by country is shown in Figure 2. As is clear from the plot, the use of the singular clitic with overt indirect plural objects (i.e., default agreement) is present in all varieties of Spanish. Perhaps the most surprising finding that stands out from these data is the fact that in no variety of Spanish is full agreement used more often than default agreement in this context.

**FIGURE 3:**
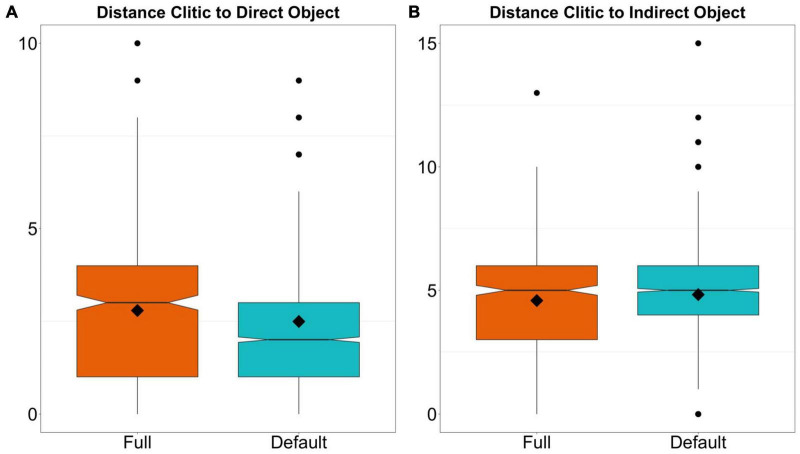
Distribution of number of syllables between the clitic and the direct object **(A)** and the clitic and the indirect object **(B)**. The rhomboid shows the mean, the line the median and the black dots are outliers.

In Figure 3, I show the distribution of the two continuous variables that measure the distance in syllables between the clitic and the direct and indirect object. The mean distance to the direct object is 2.5 and 2.8 syllables for default and full agreement, respectively. By the same token, the mean distance to the IO is 4.8 syllables for default agreement and 4.6 for full agreement. The small differences between both types of agreement suggest that distance may not be an important factor in determining clitic agreement.

**FIGURE 4:**
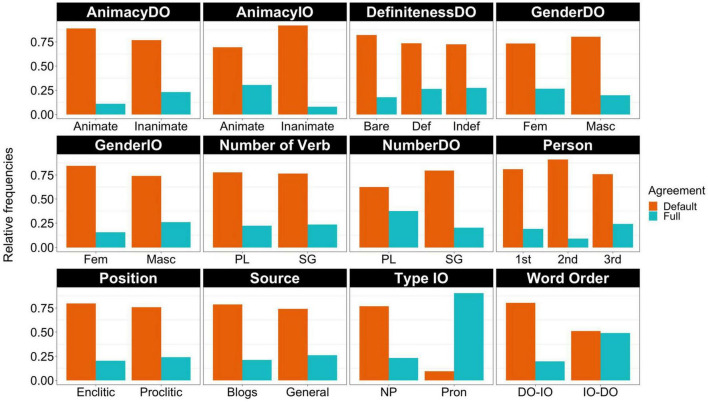
Relative frequencies of each type of agreement by predictor variable.

Figure 4 shows the relative frequencies of the remaining variables. I will focus on those that appear to show the largest differences between default and full agreement. The first difference to note is with AnimacyIO, where there appears to be a stronger preference for default agreement when the indirect object is inanimate; IOs low in animacy appear 90% of the time with default agreement but this figure goes down to 67% with animate IOs. The next variable that stands out is NumberDO, which shows that singular DOs appear 80% of the time with default agreement in contrast to 62% for plural DOs. The variable Type IO is the only variable where full agreement is more frequent for one of the levels. More concretely, it seems that pronouns reject default agreement and prefer full agreement. With lexical NPs, the pattern matches the rest of the variables in that default agreement is the preferred choice. Word order also shows a striking difference between the two levels. The unmarked word order DO-IO favors agreement 80% of the time whereas the reverse word order shows no preference and both full and default agreement appear around 50% of the time.

Based on these results, the variable Type IO was not included in the statistical analysis for the following reasons. Although one cannot say that pronouns categorically exclude default agreement, it seems that default agreement with pronouns is quite rare and goes against the trend of all the other variables because they nearly exclusively prefer full agreement. But more importantly, there is a very clear theoretical explanation of why pronouns would reject default agreement when one considers the other two contexts that disallow default agreement, namely left-dislocated IOs and non-doubled NPs. Thus, I will provide a unifying explanation for these three cases in the Discussion section.

### Conditional Inference Tree

**FIGURE 5:**
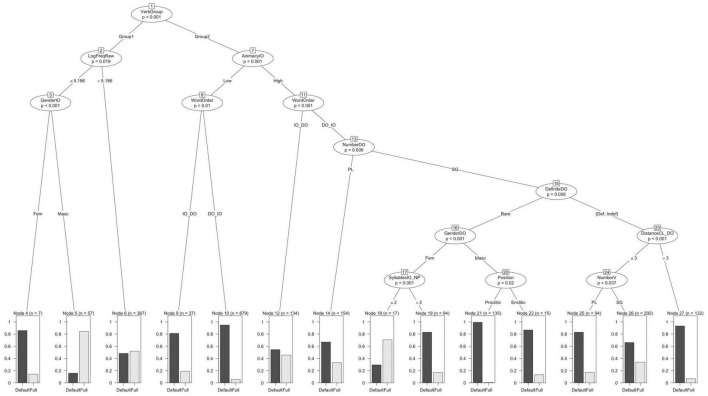
Conditional inference tree with Agreement (*defaults* vs. *full*) as dependent variable.

As mentioned above, the first model used to analyze the data is a conditional inference tree fit on all the variables except WEBSITE for the reason mentioned earlier. The tree is shown in Figure 5.

The tree model (*C*-index 0.81) divides the verbs into two groups, which I have simply called Group-1 and Group-2^15^. For Group 1, then there is a distinction between higher frequency verbs and those with lower frequency. Those with higher frequency do not show a preference for either type of agreement as shown in Node 6. For verbs in the lower frequency subset, the model finds a difference in agreement preferences depending on the gender of the IO. While masculine IOs prefer full agreement, feminine IOs show a marked preference for default agreement.

Moving on to Group-2, we find that Animacy of the IO makes a significant split in the data. With IOs low in animacy, the variable WordOrder makes an additional split, such that the DO-first word order shows a higher preference for default agreement than the IO-first word order. In contrast, with IOs that are high in animacy we find a larger number of significant splits. The first split is again with WordOrder such that the IO-first word order shows a slight preference for default agreement but this preference is much smaller compared to the DO-first word order. However, for this latter word order we find that the number of the DO creates yet another split in the data, with plural DOs showing a 0.65 vs. 0.35 relative frequency of default and full agreement, respectively. With singular DOs, we can see a number of important predictors interacting with each other but all of these favor default agreement in large proportion with one exception: singular bare feminine DOs appearing with IOs of two or fewer syllables favor full agreement.

### Bayesian Mixed-Effects Logistic Regression

Table 2 contains all the two-way and three-way interactions that make up the model reported here. I did not perform model selection on the fixed-effects to find the so-called best model because the aim was to evaluate the effect of all these predictors on clitic agreement.

**TABLE 2:** Two- and three-way interactions included in the mixed-effects logistic regression model.

Two-way interactions	Three-way interactions
NumberDO*Word Order	DefinitenessDO*GenderDO*SyllablesIO
AnimacyIO*WordOrder	DefinitenessDO*GenderDO*PositionCL
FrequencyVerb*GenderIO	DefinitenessDO*DistanceCL-DO*NumberV

The model fit is excellent with a *C-*index of 0.95 and a Bayesian *R*^2^ of 0.38 [Est.error = 0.03, CI: (0.32, 0.45)]. The classification accuracy is 0.89 [CI: (0.87, 0.90)] with a balanced accuracy of 0.78, meaning the model accurately predicts both types of agreement nearly 80% of the time.

The results of the analysis of the Bayes factors and the probability of direction are summarized in Table 3. I will focus on those parameters with a Bayes factor larger than one. The parameter with the clearest evidence of an effect is ANIMACYIO: LOW. The Bayes factor for this parameter is very large (BF > 70,000) and the probability of direction is 100%, meaning there is a 100% certainty that the direction of the effect is as it appears in the model. The parameter with the second largest amount of evidence is the two-way interaction between NUMBERDO: SG and WORDORDER: DO_IO. The Bayes factor for this interaction is 6.11 with a 99.84% certainty that the sign of the coefficient is correct. Next comes the interaction DEFINITENESSDO: DEF*POSITION: PROCLITIC, with a Bayes factor of 2.67 and with a probability of direction of 99.48%. The interaction DEFINITENESS_DO: DEF * DISTANCECL_DO has a Bayes factor of 2.08 and a 100% certainty about the direction of the effect. The last parameter I will mention is DEFINITENESSDO: BARE with a Bayes factor of nearly 1 and a probability of direction of 98.13%.

**TABLE 3:** Bayes factors (BF) and probability of direction index (PD) for each predictor variable of the mixed-effect Bayesian model.

Parameter	BF	PD
ANIMACYIO: LOW	> 7,000	100%
NUMBERDO: SG*WORDORDER: DO-IO	6.11	99.84%
DEFINITENESSDO: DEF*POSITION: PROCL	2.67	99.48%
DEFINITENESSDO: DEF*DISTANCECL_DO	2.08	100%
DEFINITENESSDO: BARE	0.93	98.13%
DEFINITENESSDO: BARE*GENDERDO: MASC	0.75	72.82%
DEFINITENESSDO: BARE*POSITION: PROCL	0.35	93.07%
WORDORDER: DO-IO	0.31	97.02%
POSITION: PROCL	0.25	95.94%
DEFINITENESSDO:DEF	0.20	89.53%
GENDERDO: MASC	0.10	95.12%
NUMBERDO: SG	0.09	81.23%
DEFINITENESSDO: BARE*GENDERDO:MASC	0.07	72.82%
DEFINITENESSDO: BARE*DISTANCECL-DO	0.02	94.77%
DISTANCECL-DO	0.00	57.18%

**FIGURE 6:**
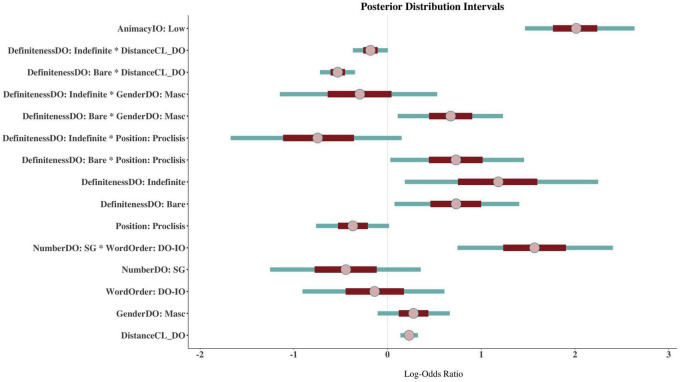
Posterior distribution intervals of the Bayesian mixed-effects model. The pink dot represents the mean, the red inner line and the teal line the 95 and 50% credible intervals, respectively.

**FIGURE 7:**
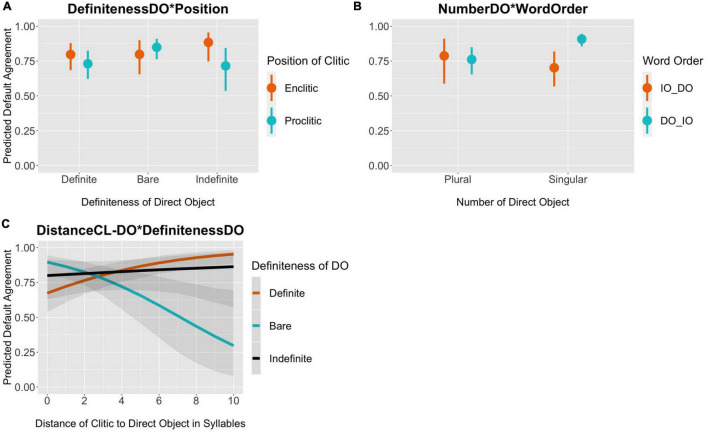
Marginal effects of interaction terms on default agreement. The y-axis represents the posterior predicted probability of default agreement.

Next, I present the results of the coefficient estimates of the model in two ways for ease of interpretation and focusing only on those parameters for which we have evidence of an effect. In Figure 6, I show the 95% posterior distribution credible intervals and in Figure 7 the conditional effects of the interactions^16^. The posterior intervals allow us to see the directionality of the effect (negative or positive) as well as the uncertainty of the mean estimate; the larger the credible interval, the less certain we can be of the true value of the coefficient estimate. The conditional effects plots make the interpretation of the interactions much easier.

In Figure 6, I will focus on the two single terms. The posterior interval of ANIMACYIO: LOW shows that indirect objects low in animacy favor default agreement [β = 2.01, CI: (1.38, 2.79)]. Although the 95% credible interval seems relatively wide, suggesting there is some variation within this parameter, the large Bayes factor reported above shows this is the parameter with the largest effect on the outcome. Similarly, the parameter DEFINITENESSDO: BARE shows a positive effect on default agreement in that bare direct objects may favor default agreement [β = 0.73, CI:(−0.05, 1.55)]. But since this predictor is involved in several interactions, we ought to be careful about its interpretation. The coefficient of this single parameter refers to bare direct objects when DISTANCECL_DO is zero, and the direct object is feminine with a clitic in an enclitic position (i.e., postverbal).

The marginal effects in Figure 7 show the predicted probability of default agreement per the model given the interaction terms. The interaction DEFINITENESSDO*POSITION shows that unlike definite and indefinite direct objects, bare direct objects with a proclitic favor default agreement (*predicted definite* = 0.73, *indef* = 0.72, *bare* = 0.85). Figure 7B shows the interaction WORDORDER*NUMBERDO. In the unmarked word order, where the DO is closer to the clitic, the predicted probability of default agreement is 0.91 when the DO is singular, compared to 0.76 when the DO is plural. The number of the DO is not predictive of agreement in the marked word order. The last interaction in Figure 7C shows the interaction DISTANCECL_DO*DEFINITENESSDO. Bare nouns have the highest probability of default agreement when they are up to 2 syllables away from the clitic. As the distance between the clitic and the DO goes up, the probability of default agreement goes down significantly reaching only 0.30 at the greatest distance of 10 syllables. With this said, there is a lot of variation as evidenced from the overlapping confidence intervals.

In short, the two models identify the animacy of the IO and the interaction between the number feature of the DO with word order as the most important variables in determining clitic agreement. However, there are some notable differences between the conditional inference tree and the mixed-effects model. In the next section, I will discuss these differences and offer an interpretation of the results in terms of attraction versus intervention effects.

## Discussion

In this section, I will first compare the results of the two models presented in the previous section. Then I will assess our hypotheses and predictions laid out in Section “Hypotheses and Predictions,” I will propose an explanation about the three contexts that disallow default agreement in Section “Default Agreement and *pro”* and round off the discussion by proposing that the probability distribution in default agreement is likely due to grammar competition.

### Model Comparison

As was shown in the previous section, both models identified as the most important variables the animacy of the IO and the interaction between the number of the DO and word order.

One difference between the two models is the status of the lexical verb variable. In the tree model, this was entered as an additional variable on a par with the other predictors, while in the mixed-effects model the verb is a random effect. While it is true that the tree model splits the verbs into two groups, it is difficult to determine whether there is some meaningful distinction between the two groups, which is why this variable is a random effect in the mixed-effects model. Having said this, one difference between the two groups is their frequency, with Group-2 having a higher (log-transformed) mean frequency than Group-1 (5.57 vs. 6.16). A Mann-Withney-Wilcoxon test confirms that this difference is statistically significant (W = 233,470, *p* < 0.001).

The other differences between the two models lie in the lower parts of the tree, namely leaves [17] and [24]. The lower the leaf, the more specific the data points under the leaf become. In other words, lower leaves reflect the existence of highly complex interactions. For example, leaf [17] refers to the number of syllables of the IO and it shows that IOs with more than two syllables show a preference for default agreement while IOs with two or fewer prefer full agreement. However, this generalization refers only to those IOs that appear with feminine DOs that are bare, singular and appearing in the unmarked word order DO-IO. In addition, the IO must be high in animacy. We can see then that the effect of the number of syllables only applies to an extremely specific subset of IOs. This six-way interaction would be impossible to capture, let alone interpret, in a regression model (but see Gries, 2020 on the limitations of classification trees in finding interactions).

However, the existence of such complex interactions also raises the question of the psychological validity of the results. That is, while it may be true that a six-way interaction may exist in a statistical sense, there is no guarantee that such complex interaction will exist in a grammatical sense. In other words, we cannot ascertain that the model found by the conditional inference tree (or any statistical model, for that matter) represents a mental grammar. We hope, and most assume without discussion, that it does but we need to be careful about our interpretation of the results and the claims that follow from them. To validate our models, we would need to assess them against human participants by, for example, comparing the predictions of the model with those of the participants, or by manipulating the predictors in the direction the model suggests and study whether participants’ responses vary as a result. With this caveat in mind, the fact that both models agree on the most prominent variables gives us a higher degree of confidence in the results.

The differences between the two models ultimately highlight their complementarity, allowing us to reach finer-grained conclusions that would be unfeasible if we only used one of them.

### Assessing the Hypotheses and Predictions

Perhaps the most important remaining question concerns the hypotheses and predictions outlined in Section “Hypotheses and Predictions.” In what follows, I will go over them one by one and assess what the results of the models say about each of them.

Hypothesis I: Default agreement can be best accounted for as a case of attraction.

Prediction 1: (summarized): If default agreement is a case of an attraction effect, then we should find an interaction between word worder and the number of the DO such that singular DOs favor default agreement in the unmarked word order (i.e., DO-IO).

This prediction was clearly borne out in both models. We saw that singular DOs in the DO-IO word order favored default agreement but the reverse word order showed no preference for either type of agreement. Moreover, we found no evidence of NUMBERDO as a main effect.

Hypothesis II: Default agreement is a by-product of the hierarchical order of the two objects.

Prediction 2: Linear distance (counted in syllables) should not be predictive of default agreement if hierarchical structure determines default agreement. In addition, we should find evidence in favor of word order such that the unmarked order DO IO should favor default agreement because, although the clitic c-commands both objects, the DO is first in the search domain of the clitic.

We found some evidence of the effect of the distance between the clitic and the DO in both models but not across the board (i.e., not as a single term). In the regression model, we found evidence of an interaction between DISTANCECL_DO and DEFINITENESSDO, such that bare DOs show a preference for full agreement when the distance between the clitic and the DO increases. We found no difference between definite or indefinite DOs. However, the tree model gives us a more nuanced picture. Here we saw that bare DOs enter into a number of interactions such that only those bare nouns that are feminine and appear with an IO of two or fewer syllables show a preference for full agreement.

On the other hand, the word order where the DO is first in the search domain of the clitic shows a preference for default agreement when the DO is singular. Thus, this prediction is neither confirmed nor rejected. It requires further research. As one reviewer points out, it may be the case that measuring distance in syllables may not be the best way to operationalize this variable. A more appropriate method might be to use the number of words between the clitic and the DO, but since in this data this number is the same across all sentences, this method cannot be implemented. Thus, I leave as an open question what role, if any, the distance between the clitic and the DO plays in clitic agreement.

Hypothesis III: The animacy of the objects will be predictive of default agreement.

Prediction 3: Inanimate indirect objects should favor default agreement because inanimate nominals in Spanish require less marking than animate ones.

This prediction was also borne out in both models. Both the tree model and the logistic regression identified animacy of the IO as a very important predictor. We can see this in the tree model because it is the second variable where the data is split. In the regression model, this is evident in the effect size of the predictor as well as the diagnostics we used to measure the amount of evidence against the null hypothesis; animacy of the IO has the largest effect size at 7.46 and a Bayes factor of over 7,000 with a probability of direction of 100%.

These results suggest that default agreement does not appear to be a case of intervention for several reasons. Under an intervention effect, we should expect default agreement whenever there is an intervening DO between the lexical IO and the clitic irrespective of the number feature of the DO, but this is not what we find in the data. In addition, the animacy feature of the IO is also key in the computation of default agreement and this has no explanation under an intervention account.

**FIGURE 8:**
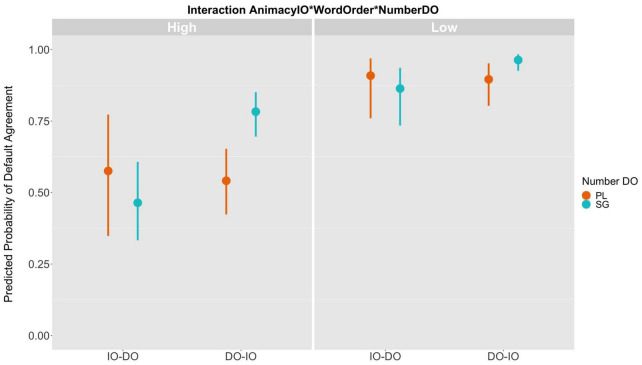
Marginal effects of the three-way interaction AnimacyIO*WordOrder*NumberDO. The y-axis represents the predicted probability of default agreement per the mixed-effects model.

However, an attraction account is not without problems. If we focus only on the effect of the interaction between word order and number of the DO, then an attraction account does seem more plausible. The clitic shows up in default agreement in a probabilistic fashion under the influence of the singular number of the DO if this is closest to it. Thus, an attraction account gives us a little more explanatory power than intervention because it can account for the role of the interaction between the number of the DO and word order, but it still cannot account for the role of the animacy of the IO. Note that the relationship between the three predictors is also quite complex as exemplified by the tree model, which shows that singular DOs prefer default agreement in the unmarked word order and with inanimate IOs. If we calculate the marginal effects of this three-way interaction from the logistic regression model, we can see, in Figure 8, that singular DOs have the highest predicted probability of default agreement at 0.96 (CI: 0.93, 0.98) in the unmarked word order with IOs low in animacy (top right panel). In this condition, they also show the least amount of variation as shown by the narrow credible interval of the predicted value.

If we analyze the predictor values that favor default agreement, it seems that most of them point toward the unmarked value with the exception of the definiteness of the DO. That is to say, default agreement appears to be most likely with DOs that are singular and masculine and IOs that are inanimate. Spanish grammar contains other phenomena where there is a distinction between marked and unmarked elements, operationalized through specificity and animacy. In differential object marking, only specific and animate objects are overtly marked for accusative case, and clitic-doubling of the direct object is also limited to highly salient NPs, namely pronouns and human beings (disregarding Porteño Spanish where DOs are more freely doubled). The puzzling aspect of default agreement is that the clitic should not enter into an agreement relationship with any features of the DO because it is a dative, not an accusative, clitic. Thus, there seem to be two major forces at play driving default agreement: the animacy of the IO coupled with the number and gender features of the DO mediated by word order. The fact that there is a difference in animacy between IOs who get full agreement and those which do not suggests that this phenomenon has some of the ingredients of differential object marking but applied to the dative object. Therefore, my preliminary conclusion is that we are in the presence of the development of a sort of differential dative marking that is masked by attraction effects of the DO. However, more research is needed to come to a more complete understanding of the interplay between these two variables and agreement of the clitic.

### Default Agreement and *pro*

In Sections “The Linguistic Phenomenon under Investigation” and “Results,” I showed three contexts where default agreement is not possible or very rare. These were with left-dislocated IOs, with pronominal IOs and with non-doubled IOs. The question that remains is why these cases disallow default agreement when it appears to be the preferred option in the unmarked clitic-doubled case.

We saw in Section “Constraints on Default Agreement” that with left-dislocated IOs, non-doubled IOs and with pronominal IOs the argument position of the verb is said to be occupied by little *pro.* The straightforward conclusion then is that default agreement is not possible with little *pro.* This licensing requirement of default agreement can be interpreted in terms of recoverability (Chomsky, 1964), which states that only recoverable deletions are permitted in the grammar. In terms of default agreement, this means that the number feature of the dative clitic may be left unspecified as long as this information can be recovered from the coreferential NP in the argument position of the verb^17,18^.

It is worth pointing out the difference between pronouns and animate lexical NPs. Given the way animacy was coded for, IOs high in animacy in this data refer to human beings with the exception of a handful of cases of “dogs” and “cats.” This strengthens the argument that the key property of the cases that do not allow default agreement must be structural since that is the major difference between lexical IOs and strong pronouns.

### The Source of the Variation

A question raised by all linguistic variation is the source of the variability. Why is it that not all cases where default agreement can occur show default agreement? Where does the probabilistic distribution we find in the data come from? A plausible answer to this question is the tension between the standard and the language acquired naturally during the language acquisition process. It is likely that vernacular Spanish no longer has agreement of the dative clitic in double object constructions with doubled lexical IOs. However, when children begin formal education, they get exposed to the standard language through textbooks, literature and writing norms, and begin to acquire the standard variety that prescriptively has obligatory agreement of the dative clitic whenever it occurs (e.g., see Rothman, 2007; [Bibr B42][Bibr B51]; for the role of schooling in the acquisition of inflected infinitives in Brazilian Portuguese). The result of higher exposure to the standard variety in school will possibly have the effect of increasing the rates of full agreement, which may be very low in pre-school children given the tendency for children to prefer a one-to-one mapping between function and form (Clark, 1987).

This proposal predicts that we should find fewer cases of default agreement in formal written Spanish that is closer to the standard. This prediction was explored by searching the Spanish version of EUR-Lex, a multilingual corpus of the official languages of the European Union made up of the legal documents of the EU. In this corpus, which contains over 635 million words, there were a total of 32 sentences matching the searches conducted for this study. Of the 32 sentences, 66% showed full agreement and 34% default agreement, which is the mirror image of the distribution in the more informal corpus used in the present study. Thus, though a much more thorough study would need to be conducted to properly assess the role of register and schooling in the realization of default agreement, the proposal that the variability in default agreement stems from the tension between the standard and the vernacular language seems on the right track.

Last, but not least, it should be mentioned that a possible limitation of the present study is the use of written data. While we found a nearly 80% rate of default agreement even with written texts, it is likely that default agreement will appear at a higher rate in spoken language where the normative pressure is lower.

## Conclusion

In this paper, we looked at a pervasive phenomenon of lack of obligatory agreement of the third-person dative clitic in double-object constructions with doubled IOs. By analyzing corpus data with two complementary statistical models, we found that four variables seem to be the most influential in the agreement variation of the clitic, namely animacy of the IO, the interaction between number of the DO and word order, and the definiteness of the DO. I argued that the results of the statistical analyses are incompatible with an intervention account because this type of phenomenon is not sensitive to semantic features of the intervening element or to the true controller of agreement. In contrast, I proposed that the data is best analyzed as the interplay between attraction and the morphosyntax of the unmarked. In Spanish, this results in IOs showing a sort of differential dative marking where inanimate IOs show a preference for default agreement and attraction effects from the singular DO in the unmarked word order.

Furthermore, I also showed that default agreement is limited by structural constraints such that default agreement can only take place when the argument position of indirect object is filled with a lexical NP; little *pro* cannot license default agreement.

In addition, I proposed that the overall variation between full and default agreement can be understood as the tension between the standard and the vernacular language, which results in two competing grammars with different probability distributions in speakers’ minds.

The findings in this paper raise a number of new research questions concerning the role of attraction versus intervention in agreement variation and the nature of morphosyntactic agreement more generally. More importantly, the results also make clear predictions that can be tested empirically to help advance our understanding of linguistic variation and morphosyntactic processes.

## Data Availability Statement

The datasets and code for the statistical analysis supporting the present findings of this article are publicly available via doi: 10.7910/DVN/94I9DX.

## Author Contributions

GG extracted the data from the corpus, annotated it, ran the statistical analysis, and wrote the manuscript.

## Conflict of Interest

The author declares that the research was conducted in the absence of any commercial or financial relationships that could be construed as a potential conflict of interest.

## Publisher’s Note

All claims expressed in this article are solely those of the authors and do not necessarily represent those of their affiliated organizations, or those of the publisher, the editors and the reviewers. Any product that may be evaluated in this article, or claim that may be made by its manufacturer, is not guaranteed or endorsed by the publisher.

## Footnotes

1 These examples come from Sketch Engine’s esTenTen18 corpus on www.sketchengine.eu. There are over 6,500 examples of this pattern with *importante (1,176), excelente (887), diferente (363), consciente (257)* and *frecuente* (172) as the top five adjectives ending in –*nte.* An additional 2,258 examples come from adjectives ending in *–l*, *–z* or –*e*, the top five being *fácil* (644), *capaz* (631), *difícil* (421), *grande* (209) and *fundamental* (149).

2 This agreement mismatch is not limited to predicative position of the adjective. It is also common in attributive position preceding a plural noun as in (i).

3 The dataset and R-Markdown with the code of the statistical analysis is available on https://tinyurl.com/eby9s3t8. At the moment, it is only available for reviewers *via* this private link and all materials have been anonymized.

4 The country below each example refers to the country tagged by the corpus. The number is the ID of the sentence in the dataset.

5 The sentence in (10a′) should be interpreted as the reply to the question in (10a), not as a standalone sentence.

6 An exploratory search in corpus del Español and esTenTen18 on Sketch Engine confirms this. 4.6% (*N* = 2,992) and 4.3% (*N* = 10,155) of clauses of the form “A los| las N* le| les GUSTAR” appear with default agreement, respectively.

7 The syntactic constraints on default agreement demonstrate that this phenomenon cannot be reduced to a phonological issue of s-dropping or s-aspiration, which are common processes in Spanish.

8 Within generative linguistics, movement is a syntactic operation whereby lexical objects may originate, and be interpreted, in one position but appear in a higher position in the phonetic or surface form. In clitics, this is evident, for example, in that clitics are interpreted as objects of the verb but they must appear pre-verbally when the verb is finite (see Anagnostopoulou, 2017 for a discussion on the role of movement in clitic doubling).

9 Note that word order in itself cannot distinguish between linear order or hierarchical structure because linearly closer and hierarchically closer coincide in this context as whichever object is first will satisfy both conditions. As stated in Prediction 1, word order can distinguish between attraction and intervention in connection with the number feature of the DO. Ultimately, Prediction 2 says that if linear order is not significant in a statistical sense but word order is significant then we have more evidence in favor of an attraction account.

10 A reviewer (who eventually withdrew) questions the validity/accuracy of referring to the United States as a Spanish-speaking country claiming that Spanish is not an official language in the United States nor do the majority of the people speak Spanish in this country. First of all, the constitution of the United States does not designate any specific language as official to the United States, not even English. More importantly, though, denying the existence of over 35 million Spanish speakers in the United States simply because they do not speak the majority language seems both unfair and dangerous. The description of the corpus is taken verbatim from Mark Davie’s description of the corpus and numerous authors refer to the United States as a Spanish-speaking country, because it is. In the *Routledge Handbook of Spanish as a Heritage Language* (Potowski and Muñoz-Basols, 2018), Jenkins (2018: 53) states *“The United States has a formidable presence in the Spanish-speaking world. Of the 50.5 million Hispanics that live in the United States, 35-4 million of them speak Spanish. That figure ranks it as the fifth-largest Spanish-speaking country in the world.”*

11 The same reviewer is concerned that the United States data was included in the analysis claiming that Spanish speakers in the United States are mostly heritage or L2 speakers. The data from the United States made up 8% of the total data and there were no statistical significant differences across countries, as all of them have similar rates of default agreement as shown in the Results section. If the data from the United States had behaved differently from the rest of the countries, then I would have considered removing these data or paying closer attention to it, but since this was not the case, the data was kept.

12 There were nevertheless only 14 and 6 examples of *dog* and *cat*, respectively. The rate of default agreement was 70% with human-only and 90% with *dog* and *cat* IOs.

13 The equivalence between the probability of direction (Pd) and the two-sided *p*-value is given by the formula *p* = 2 × (1–Pd).

14 A table with the Bayes factors of the full model is available in the Appendix in Table 1.

15 Group-1: *agradecer* “thank,” *añadir* “add,” *aplicar* “apply,” *asegurar* “ensure,” *asignar* “assign,” *besar “*kiss,” *buscar* “look for,” *caer* “fall,” *callar* “keep quiet,” *cargar* “carry,” *causar* “cause,” *cerrar* “close,” *colocar* “put,” *componer* “compose,” *comprar* “buy,” *comunicar* “communicate,” *conceder* “concede,” *conferir* “confer,” *congelar* “freeze,” *conseguir* “achieve,” *construir* “build,” *contar* “tell,” *crear* “create,” *cubrir* “cover,” *decir* “say,” *dejar* “leave,” *descontar* “discount,” *dormir* “sleep,” *efectuar* “perform,” *encomendar* “request,” *endosar* “endorse,” *enseñar* “teach,” *entregar* “deliver,” *exigir* “demand,” *explicar* “explain,” *facilitar* make easy,” *fallar* “fail,” *formar* “make up,” *garantizar “*guarantee,” *generar* “generate,” *implicar* “imply,” *imputar* “impute,” *indicar* “indicate,” *informar* “inform,” *instalar “install,” interiorizar* “to acquaint with,” *“ir’go,” jalar “*pull,” *lavar* “wash,” *lazar “*lasoo,” *leer* “read,” *legalizar* “legalize,” *llamar* “call,” *llegar* “arrive,” *llenar* “fill,” *mandar* “send,” *mostrar* “show,” *negar* “deny,” *obsequiar* “gift,” *ocasionar* “cause,” *ocultar* “hide,” *ofrecer* “offer,” *otorgar* “grant,” *pagar* “pay,” *parar* “stop,” *partir* “break,” *pedir* “ask for,” *permitir* “allow,” *practicar* “practise,” *preguntar* “ask,” *presentar* “introduce,” *prohibir* “prohibit,” *prometer* “promise,” *propiciar* “encourage,” *reconocer* “acknowledge,” *recorder* “remember,” *recortar* “cut down,” *refrescar* “freshen up,” *repartir* “hand out/round,” *reportar* “report,” *resguardar* “shelter,” *respetar* “respect,” *retribuir* “give back,” *revisar* “double check,” *salvar* “save,” *significar* “mean,” *solicitar* “request,” *soltar* “let go of,” *temblar* “shake,” *transmitir* “convey.” Group-2: *abrir* “open,” *achacar* “impute,” *agarrar* “grab,” *agregar* “add,” *ahorrar* “save,” *alquilar* “rent,” *aportar* “contribute,” *arrancar* “rip out,” *arrebatar* “snatch,” *asestar* “deal,” *asistir* “assist,” *atar* “tie,” *atribuir* “attribute,” *aumentar* “increase,” *ayudar* “help,” *bajar* “decrease,” *brindar* “give,” *cambiar* “change,” *ceder* “give/yield,” *celebrar* “celebrate,” *cercenar* “curtail,” *chupar* “suck,” *cobrar* “cobrar,” *coger* “grab/get,” *comer* “eat,” *condonar* “condone,” *considerar* “consider,” *corresponder* “correspond,” *cortar* “cortar,” *costar* “costar,” *cuidar* “take care of,” *cumplir* “comply with,” *dar* “give,” *deber* “owe,” *declarar* “declare,” *decretar* “decrete,” *dedicar* “dedicate,” *deparar* “set,” *desear* “desire/wish,” *despedazar* “tear up,” *destrozar* “destroy,” *devolver* “return,” *dictar* “dictate,” *dirigir* “direct,” *doler* “hurt,” *durar* “last,” *echar* “dismiss,” *emitir* “emit,” *encontrar* “find,” *enganchar* “hook up,” *engordar* “fatten,” *enmendar* “fix,” *enviar* “send,” *escribir* “write,” *espetar* “surprise, *estrechar* “shake hands,” *evitar* “avoid,” *extender* “extend,” *extraer* “extract,” *faltar* “lack,” *fijar* “stick,” *ganar* “win,” *gritar* “scream,” *hacer* “make/do,” *hormiguear* “itch,” *impedir* “prevent,” *imponer* “impose,” *importar* “care about,” *infundir* “ingrain,” *introducir* “introduce,” *jugar* “play,” *lamer* “lick,” *limpiar* “clean,” *llevar* “take,” *mamar* “suckle,” *marcar* “mark,” *matar* “kill,” *meter* “put,” *minar* “mine,” *morder* “bite,” *mover* “move,” *ocurrir* “occur,” *organizar* “organise,” *parecer* “seem,” *pasar* “happen/pass,” *patear* “kick,” *pavimentar* “pave,” *pegar* “hit/stick,” *perder* “lose,” *perdonar* “forgive,” *pesar “*weigh,” *plantear* “raise/propose,” *platicar* “talk,” *poner* “put,” *preparar* “prepare,” *prestar* “lend,” *producir* “produce,” *propinar* “tip,” *proponer* “propose,” *proporcionar “*provide,” *proveer* “provide,” *pulverizar* “grind,” *quedar* “fit,” *quitar* “remove,” *realizar* “do,” *recuperar “*recover,” *regalar* “gift,” *regresar* “return,” *remover* “remove,” *render* “yield,” *resolver* “solve,” *restar* “deduct,” *robar* “steal/rob,” *romper “*break,” *sacar* “take out,” *seguir* “continue,” *señalar* “point,” *sentir* “feel,” *ser* “be,” *server* “serve,” *simplificar* “simplify,” *solucionar* “solve,” *subir* “increase,” *subvencionar* “subsidize,” *sugerir “*suggest,” *suministrar* “provide,” *suponer* “suppose,” *tener* “have,” *tirar* “throw,” *tocar “*touch,” *tomar* “take,” *torcer* “twist,” *traer* “bring,” *tranferir* “transfer,” *valer* “be worth,” *vedar* “veto,” *vender* “sell,” *ver* “see,” *violentar “*stir up,” *volar* “fly.”

16 A table with coefficients and credible intervals of the full model is available in Table 2 in the Appendix.

17 Note that this conclusion is independent of whether *pro* or a copy of the clitic occupies the argument position of the verb. The important point of this conclusion is that default agreement is incompatible with empty elements in argument position because the deleted number feature cannot be recovered.

18 One may wonder why deleted features could not be recovered from context, for example from the left-dislocated IO when there is one. The key distinction here is the difference between a formal and a functional understanding of recoverability (I thank Gillian Ramchand for this observation). A formal account is one which derives language structure from a set of principles that stem from grammar-internal primitives. In contrast, a functional explanation resorts to issues in communication, which are said to be common across cultures and/or to the general human cognitive system (Newmeyer, 2017). My argument is intended to be understood within a formal approach. Thus, my proposal is that the grammar does not allow features to be recovered from elements that are not in argument position. Of course, context always makes available features that could be interpreted but the grammar may not allow for this to happen. A clear example is null subjects. Languages that do not allow null-subjects could recover the reference of the subject from the context in many cases and yet we know this does not matter; if a language does not allow null-subjects then context becomes irrelevant for subject realization.

## Appendix

**TABLE A1:** Bayes factors of full model.

Parameter	BF	Parameter	BF	Parameter	BF	Parameter	BF
Animacy-IO: Low DefiniteDO: Def *	663.14	GenderDO: Masc*Position: Proclitic	0.392	DefiniteDO: Bare*NumberV: PL	0.146	DefiniteDO: Bare*Syllables-IO	0.02
Position: Proclitic	5.55	GenderDO: Masc*DefiniteDO: Bare	0.261	GenderDO: Masc	0.125	GenderDO: Masc*Syllables-IO	0.018
NumberDO: SG* WordOrder: DO-IO	4.43	GenderDO: Masc*DefiniteDO: Def*Syllables-IO	0.21	NumberDO: SG	0.11	DefiniteDO: Bare*DistanceCL_DO*NumberV: PL	0.015
GenderDO: Masc* DefiniteDO: Def	1.49	Position: Proclitic	0.189	Verb Freq*Gender-IO: Masc	0.083	DefiniteDO: Def*DistanceCL_DO*NumberV: PL	0.015
DefiniteDO: Def* DistanceCL_DO	1.23	DefiniteDO: Def	0.186	Gender-IO: Masc	0.058	Syllables-IO	0.007
WordOrder: DO-IO	1.14	WordOrder: DO-IO*Animacy-IO: Low	0.186	GenderDO: Masc*DefiniteDO: Bare*Syllables-IO	0.037	DistanceCL_DO*NumberV: PL	0.005
DefiniteDO: Bare	0.475	GenderDO: Masc*DefiniteDO: Def*Position: Proclitic	0.167	DefiniteDO: Bare*DistanceCL_DO	0.036	DistanceCL_DO	2.38E- 04
DefiniteDO: Def* NumberV: PL	0.45	NumberV: PL	0.166	Verb Freq	0.035		
DefiniteDO: Bare *		GenderDO: Masc*DefiniteDO: Bare					
Position: Proclitic	0.401	* Position: Proclitic	0.161	DefiniteDO: Def*Syllables-IO	0.026		

**TABLE A2:** Final model summary.

	Estimate	Est.Error	l–95% CI	u-95% CI	Rhat	Bulk_ESS	Tail_ESS
**Group-Level Effects: Country (Number of levels: 21)**							
sd (Intercept)	0.19	0.13	0.01	0.49	1	3,157	5,432
sd (NumberDO: SG)	0.34	0.18	0.03	0.73	1	2,497	3,038
sd (AnimacyIO: Low)	0.25	0.20	0.01	0.75	1	4,703	5,906
**LexicalDO (Number of levels: 712)**							
sd (Intercept)	0.71	0.28	0.09	1.21	1.01	568	712
sd (NumberDO: SG)	0.48	0.31	0.02	1.12	1	610	1,857
Sd (AnimacyIO: Low)	1.33	0.46	0.41	2.27	1	1,415	1,548
**NounIO (Number of levels: 805)**							
sd (Intercept)	0.74	0.18	0.39	1.12	1.01	1,543	2,507
sd (NumberDOSG)	0.34	0.24	0.01	0.88	1.01	1,164	2,332
**Verb (Number of levels: 230)**							
sd (Intercept)	0.58	0.24	0.08	1.04	1	1,114	1,523
sd (NumberDO: SG)	0.44	0.29	0.02	1.05	1	1,233	3,435
sd (AnimacyIO: Low)	0.53	0.39	0.02	1.45	1	2,352	4,852
**Population-level effects:**							
Intercept	−0.33	0.52	−1.35	0.71	1	7,933	8,829
AnimacyIO: Low	2.01	0.36	1.38	2.79	1	3,176	5,806
DefiniteDO: Bare	0.73	0.40	−0.05	1.55	1	9,070	8,659
DefiniteDO: Indef	1.18	0.63	−0.01	2.47	1	7,936	8,169
Position: Proclitic	−0.37	0.24	−0.84	0.08	1	8,228	8,943
NumberDO: SG	−0.45	0.49	−1.42	0.51	1	6,539	7,919
WordOrder: DO_IO	−0.14	0.46	−1.05	0.75	1	6,951	7,626
GenderDO: Masc	0.28	0.23	−0.19	0.73	1	8,019	7,969
DistanceCL_DO	0.23	0.06	0.12	0.34	1	6,891	8,226
**DefiniteDO: Bare*Position:**							
Proclitic	0.73	0.43	−0.11	1.58	1	7,391	8,738
**DefiniteDO: Indef*Position:**							
Proclitic	−0.75	0.55	−1.84	0.31	1	9,682	9,438
**NumberDO: SG*WordOrder:**							
DO_IO	1.57	0.50	0.60	2.58	1	6,505	7,570
DefiniteDO: Bare*GenderDO: Masc	0.67	0.34	0.00	1.34	1	7,382	8,198
**DefiniteDO: Indef*GenderDO:**							
Masc	−0.3	0.51	−1.32	0.69	1	8,504	8,831
DefiniteDO: Bare*DistanceCL_DO	−0.53	0.11	−0.76	−0.31	1	7,126	8,246
**DefiniteDO:**							
Indef*DistanceCL_DO	−0.18	0.11	−0.41	0.04	1	8,404	7,811
